# Efficient Solar-Induced Photoelectrochemical Response Using Coupling Semiconductor TiO_2_-ZnO Nanorod Film

**DOI:** 10.3390/ma9110937

**Published:** 2016-11-22

**Authors:** Nur Azimah Abd Samad, Chin Wei Lai, Kung Shiuh Lau, Sharifah Bee Abd Hamid

**Affiliations:** Nanotechnology & Catalysis Research Centre (NANOCAT), 3rd Floor, Block A, Institute of Postgraduate Studies (IPS), University of Malaya, 50603 Kuala Lumpur, Malaysia; nurazimah@um.edu.my (N.A.A.S.); lauks@um.edu.my (K.S.L.); sharifahbee@um.edu.my (S.B.A.H.)

**Keywords:** TiO_2_-ZnO composite thin film, ZnO nanorod, TiO_2_ nanoparticles, photocurrent response

## Abstract

Efficient solar driven photoelectrochemical (PEC) response by enhancing charge separation has attracted great interest in the hydrogen generation application. The formation of one-dimensional ZnO nanorod structure without bundling is essential for high efficiency in PEC response. In this present research work, ZnO nanorod with an average 500 nm in length and average diameter of about 75 nm was successfully formed via electrodeposition method in 0.05 mM ZnCl_2_ and 0.1 M KCl electrolyte at 1 V for 60 min under 70 °C condition. Continuous efforts have been exerted to further improve the solar driven PEC response by incorporating an optimum content of TiO_2_ into ZnO nanorod using dip-coating technique. It was found that 0.25 at % of TiO_2_ loaded on ZnO nanorod film demonstrated a maximum photocurrent density of 19.78 mA/cm^2^ (with V vs. Ag/AgCl) under UV illumination and 14.75 mA/cm^2^ (with V vs. Ag/AgCl) under solar illumination with photoconversion efficiency ~2.9% (UV illumination) and ~4.3% (solar illumination). This performance was approximately 3–4 times higher than ZnO film itself. An enhancement of photocurrent density and photoconversion efficiency occurred due to the sufficient Ti element within TiO_2_-ZnO nanorod film, which acted as an effective mediator to trap the photo-induced electrons and minimize the recombination of charge carriers. Besides, phenomenon of charge-separation effect at type-II band alignment of Zn and Ti could further enhance the charge carrier transportation during illumination.

## 1. Introduction

ZnO nanostructure is a rapidly developing metal oxide. The dynamic design and promising functional properties attract momentous scientific interest. With its vast nano-architecture, ZnO nanorod shape is the most studied photocatalyst in photoelectrochemical (PEC) response. As mentioned above, noticeable limitation, specifically its poor solar illumination absorption and rapid recombination charge carrier losses, hinder further practice in electronic application. In general, copious researches proved that performance of ZnO nanorod photocatalyst (solar illumination absorption and recombination of charge carrier losses) could be improved by coupling with another semiconductor photocatalyst [1,2]. Therefore, the objective of this research work is to study the PEC system performance from TiO_2_-ZnO nanocomposite photoelectrode under ultraviolet (UV) and solar illumination. The novelty lies via the simple combination of electrodeposition and dip-coating that have not been approached by any other researcher (Table 1).

ZnO nanorods can be formed from sol-gel method [3,4], hydrothermal method [5,6,7,8,9,10], solvothermal method [11,12,13,14], chemical vapor deposition (CVD) method [15,16,17,18], atomic layer deposition (ALD) method [19,20,21,22], electrodeposition method [23,24,25,26], and other methods. However, due to several advantages, electrodeposition method has been chosen for the formation of ZnO nanorods in this research work. The advantages of electrodeposition method are that it is simple, quick, and economic; able to control the crystallization of ZnO nanorods [27]; its low temperature condition and low equipment cost; and the precise controllability and repeatability of nanostructures [28]. There are a few unique elements in electrodeposition process used in this research work. First, the rarely-used zinc substrate, which helps improve the electron movement from TiO_2_-ZnO to external circuit. Second, it is template-free and seed layer-free. Third, no additional acid was applied in controlling electrolyte pH during electrodeposition process. This combination brought the difference of electrodeposition method in this research work from previous work. Further explanation is included in Section 3.1.

Furthermore, the modification of binary oxide that arises from the enrichment of second oxide on primary oxide diminishes radiationless transfer of the photon energy absorbed by second oxide [29]. In addition, Anpo et al. have proven that the enhancement of the photocatalytic activity of the TiO_2_ species in the primary oxide have a lower Ti content [29]. The coupling of two semiconductors with appropriate energy, CB and VB, can reduce the recombination of e^−^/h^+^ pairs due to the transfer of carriers from one semiconductor to the other. Furthermore, depending on the band-gap energy of the semiconductor used, the composite can be activated in the visible region [30]. The interfacial potential gradient, corresponding to the energetic position, plays a role by providing better charge carrier transportation, and charge carrier separation can be achieved by modification of core photocatalyst [31,32].

When the core photocatalyst coupled with another semiconductor is activated by illumination, electrons are injected from the semiconductor with a more negative conduction band (CB) level to the positive one, while holes are transferred from the semiconductor with a more positive valence band (VB) level to the negative one. Thus, separation of charge carriers could be achieved; consequently, the lifetime of the charge carriers and the efficiency of the interfacial charge transfer to water increase significantly [7]. Details of hybrid TiO_2_-ZnO formation based on different method from past researchers are summarized in Table 1. Optimum amount of incorporated TiO_2_-based ZnO formation results in extended lifetimes of charge carriers and suppression of the recombination losses effectively. The modification of ZnO could lead to higher photocatalytic activity than ZnO itself. Besides, the improvement in light absorption occurred from UV region to visible region.

## 2. Results and Discussion

### 2.1. Morphological Studies

**Figure 1:**
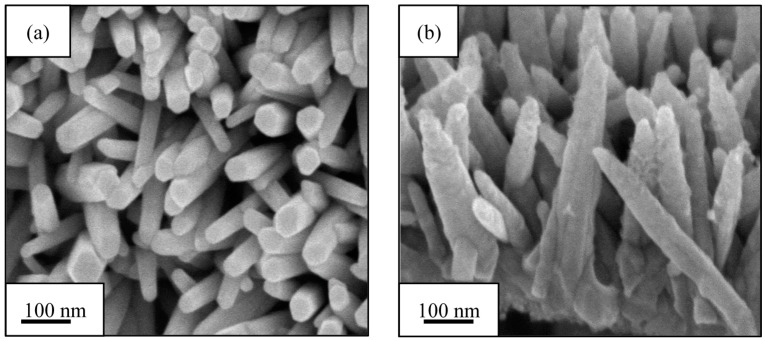

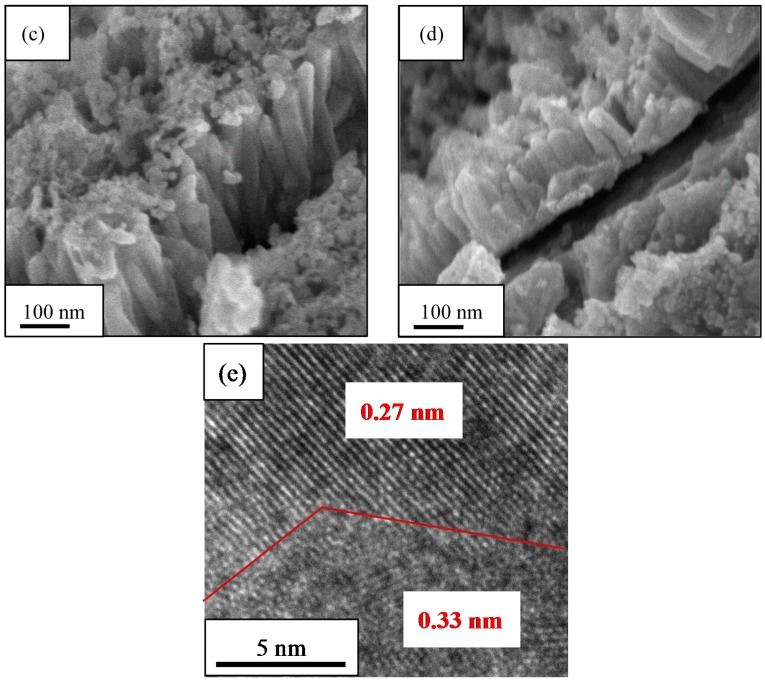
FESEM images with 100 k magnification: (**a**) ZnO; (**b**) 0.25 at % TiO_2_-ZnO; (**c**) 0.50 at % TiO_2_-ZnO; and (**d**) 1.0 at % TiO_2_-ZnO; (**e**) HRTEM image for TiO_2_-ZnO.

**Figure 2:**
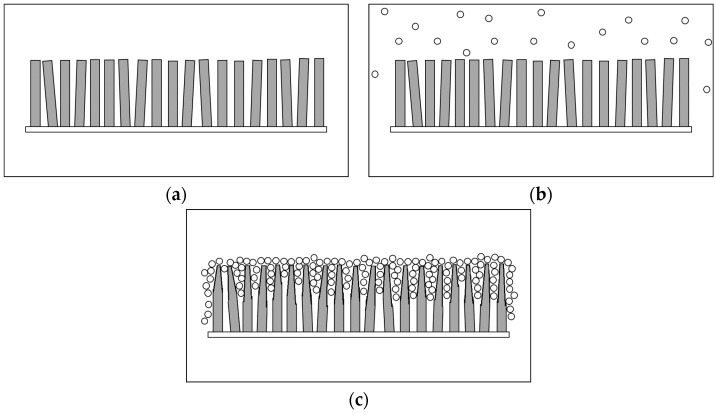
The schematic diagram of dip-coating method for the formation of TiO_2_ loaded-ZnO: (**a**) ZnO nanorods; (**b**) dip-coating process; and (**c**) TiO_2_ loaded-ZnO and followed by calcination process at 400 °C.

After electrodeposition and dip-coating methods, TiO_2_-ZnO can be found on both sides of the electrode. Each additional dip-coating cycle produced a small change in TiO_2_-ZnO thin film morphology (Figure 1). Meanwhile, Figure 2 shows the schematic diagram of dip-coating method for the formation of TiO_2_ loaded-ZnO. Average compositional ratio for ZnO, one-cycle dip-coating TiO_2_-ZnO, two-cycle dip-coating TiO_2_-ZnO, and three-cycle dip-coating TiO_2_-ZnO are shown in Table 2 using EDX spectroscopy analysis. From here, samples are named after the titanium atomic percentage (at %). One-cycle dip-coating TiO_2_-ZnO, two-cycle dip-coating TiO_2_-ZnO, and three-cycle dip-coating TiO_2_-ZnO are named 0.25 at %, 0.50 at %, and 1.0 at %, respectively. FESEM images show that all samples were very nearly vertically aligned and were of the average length, diameter and aspect ratio shown in Table 3. In addition, Figure 1a–d shows a decrease in the length and diameter with the increase in dip-coating cycles. This result is attributed to the etching phenomenon by the TiO_2_ solution which was in an acidic (pH 1–3) solution to maintain the dispersion of TiO_2_. From the HRTEM result, there was a boundary that split the two different materials (Figure 1e). This was confirmed by the lattice spacing by each material: 0.27 nm (ZnO (002)) and 0.33 nm (TiO_2_). In addition, the existence of the two different materials could be recognized by the arrangement of atoms in different directions.

### 2.2. Crystallinity Studies

**Figure 3:**
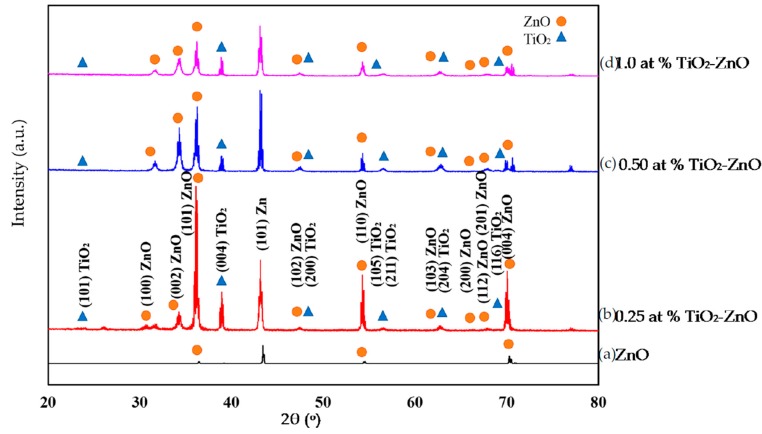
XRD pattern of (**a**) ZnO; (**b**) 0.25 at % TiO_2_-ZnO; (**c**) 0.50 at % TiO_2_-ZnO; and (**d**) 1.0 at % TiO_2_-ZnO.

Figure 3 shows the XRD pattern for 0.25 at % TiO_2_-ZnO, 0.5 at % TiO_2_-ZnO, 1.0 at % TiO_2_-ZnO and ZnO thin film. In Figure 3b–d, peaks of TiO_2_ and ZnO could be observed. TiO_2_ is denoted by 25.4° (101), 37.3° (103), 38.2° (004), 38.9° (112), 48.3° (200), 54.0° (105), 55.5° (211), and 63.2° (204) (ICDD 01-073-1764). Meanwhile, ZnO is denoted by 31.9° (100), 34.6° (002), 36.2° (101), 47.4° (102), 56.4° (110), 62.9° (103), 68.1° (112), and 69.0° (201) (ICDD 01-080-0074). Increasing the dip-coating cycle produced a lower intensity of FWHM of ZnO. This is because the TiO_2_ volume started to increase. Moreover, TiO_2_ loaded on ZnO nanorod film showed that no other elements exist. The quantification 0.25 at % TiO_2_-ZnO, 0.5 at % TiO_2_-ZnO, and 1.0 at % TiO_2_-ZnO were noted as 25%–75%, 34%–66%, and 33%–67%, respectively. Meanwhile, ZnO can be indexed to wurtzite ZnO (ICDD 00-036-1451) without any impurity peaks (Figure 3a). Good crystallinity can be seen from the sharp peaks of ZnO prior to the dip-coating method for TiO_2_-ZnO.

**Figure 4:**
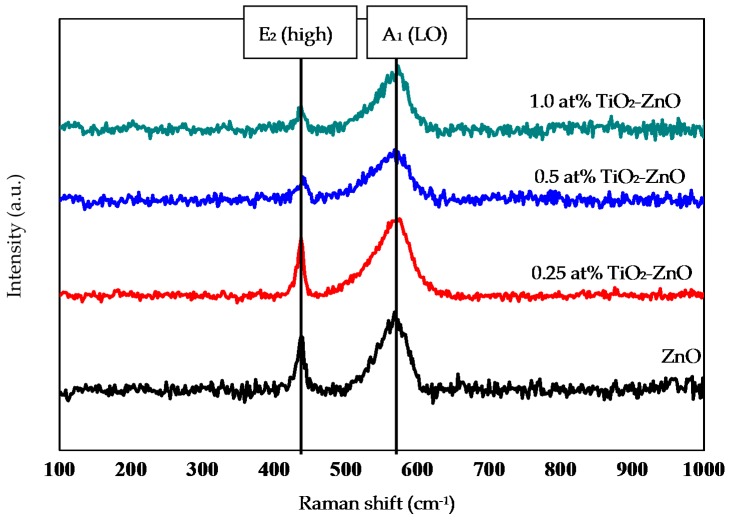
Raman scattering of ZnO, 0.25 at % TiO_2_-ZnO, 0.5 at % TiO_2_-ZnO, and 1.0 at % TiO_2_-ZnO.

Raman analysis was used to determine and understand the structural changes of ZnO and TiO_2_ upon increasing the dip-coating cycle. However, Figure 4 shows no signature of the TiO_2_ substance, with the Raman peaks mainly belonging to wurtzite ZnO. This might be due to the very small amount of TiO_2_ and to the scattering spectra that could be negligible as they were too small to be seen. These thin films were still weak in stoichiometric ZnO due to the dominance of the E_1_ (LO) and A_1_ (LO) modes (570–585 cm^−1^) for all samples compared to the E_2_ (high) mode 438 cm^−1^. This can be explained by the oxygen atom deficiency that was represented by these two modes (E_1_ (LO) and A_1_ (LO) mode (570–585 cm^−1^)) and with the existence of the Zn element from the Zn substrate [38,39]. Increasing the dip-coating cycle produced a lower E_2_ (high) mode, which was attributed to the depreciation of the perfect crystal structure wurtzite ZnO. Meanwhile, as mentioned above, there was a dominance of the E_1_ (LO) and A_1_ (LO) mode (570–585 cm^−1^) for all samples compared to the E_2_ (high) mode 438 cm^−1^. However, an increase in the dip-coating cycle produced a slight depreciation in the E_1_ (LO) and A_1_ (LO) modes. Increasing the dip-coating cycle decreased the absorption of light, producing slightly lower Raman spectra. This argument is supported by reflectance spectra under section 2.5 Optical Properties (Figure 15). The small shift of spectra was probably due to the optical phonon confinement, a defect or impurity in the nanocrystal, laser irradiation heating, or the tensile strain effect [40,41].

**Figure 5:**
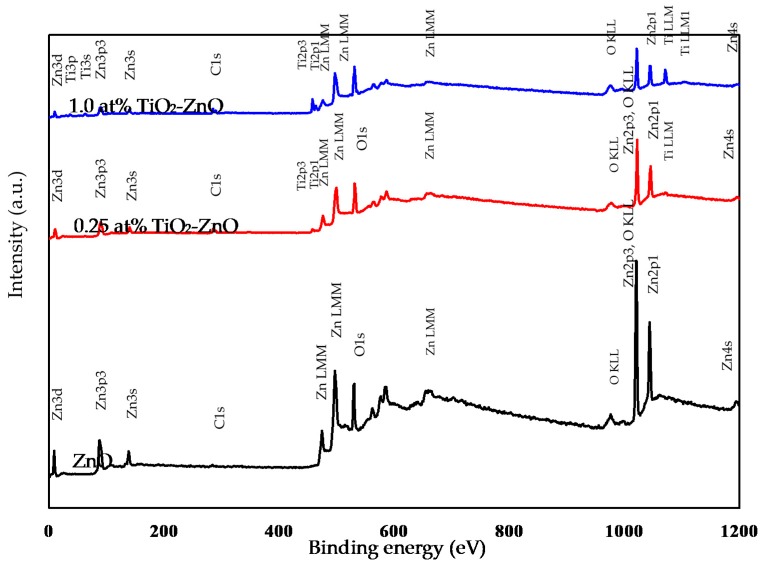
Full XPS survey spectra of ZnO, 0.25 at % TiO_2_-ZnO, and 1.0 at % TiO_2_-ZnO.

**Figure 6:**
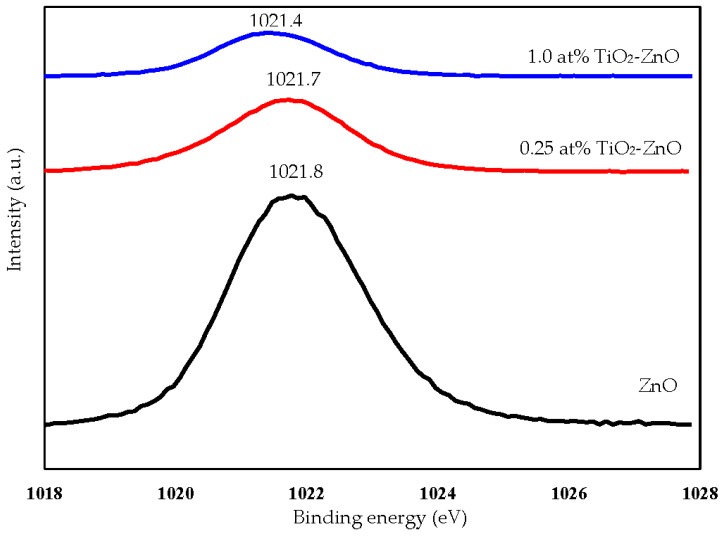
XPS spectra Zn2p of ZnO, 0.25 at % TiO_2_-ZnO, and 1.0 at % TiO_2_-ZnO.

For a further understanding of the elements and chemical interaction of the TiO_2_-ZnO interface, a XPS analysis was carried out for samples ZnO, 0.25 at % TiO_2_-ZnO and 1.0 at % TiO_2_-ZnO. XPS survey spectra confirmed that TiO_2_ was successfully deposited onto the ZnO film. The elements Zn, Ti, C, and O existed in the TiO_2_-ZnO nanorods (Figure 5). The XPS results showed that the Ti peaks increased with the dip-coating cycle. This is in accordance with the EDX results. One peak of Zn2p_3/2_ was detected at binding energy 1021 ± 1.0 eV (Figure 6) and this matched the CAS registry No. 1314-13-2, referring to National Institute of Standards and Technology (NIST), an agency of the U.S. Department of Commerce [42]. From this, the sample with the formula ZnO is classed as a catalyst and an oxide with the line designation 2p_3/2_ and a related-binding energy of 1021 ± 1.0 eV [43]. With the increase in the dip-coating cycle, the binding energy of Zn2p_3/2_ shifted to a lower binding energy (Figure 6). The difference in binding energies is attributed to the change of charge transfer from Zn^2+^ to O^2−^. In addition, previous research has shown that oxygen deficiency is the main factor in the decrease in binding energy [44,45]. This argument was supported by the Zn2p and O1s binding energies were shifted to lower binding energies after the coating method. The XPS results showed a decrease in the Zn/O ratio for TiO_2_ loaded on ZnO nanorod film as compared to the ZnO itself.

**Figure 7:**
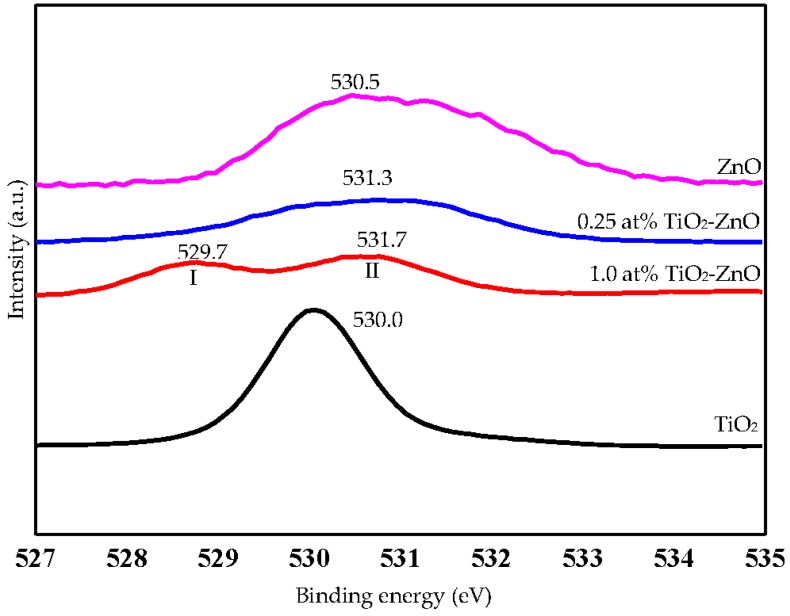
XPS spectra O1s of TiO_2_, 0.25 at % TiO_2_-ZnO, 1.0 at % TiO_2_-ZnO, and ZnO.

Referring to Zhang et al. and Al-Gaashani et al., O1s binding energies at 530.4 eV, 531.4 eV, and 532.4 eV are O^2−^ species in the lattice (O_L_), oxygen vacancies and defects (O_v_), and chemisorbed or dissociated (O_c_) oxygen species, respectively [46,47]. Meanwhile, the O1s binding energies associated in all samples in this research work are 530.5 eV (ZnO), 529.7 eV and 531.7 eV (1.0 at % TiO_2_-ZnO), 531.7 eV (0.25 at % TiO_2_-ZnO), and 530.0 eV (TiO_2_). It can be clearly seen that all O1s curves were asymmetric; therefore, both lines were fitted with two Gaussian peaks (I and II) (Figure 7 (1.0 at % TiO_2_-ZnO)). Peak I of O1s was located in the lower binding energy, as compared to peak II. Peak I was assigned for the O^2−^ ions of the Zn-O bonding at the crystal lattice (O_L_) [46,48]. For peak II (1.0 at % TiO_2_-ZnO), the location of 531.7 eV is located in between oxygen vacancies defect (531.4 eV) and existence of hydroxyl group (532.4 eV). From the EDX result, it was confirmed that O at % is reduced with the increase in the dip-coating cycle (Table 2). However, the existence of –OH group is accepted as Zn-OH formed before the formation of ZnO. These hydroxyl groups helped to prevent the recombination of electron–holes [44,49].

**Figure 8:**
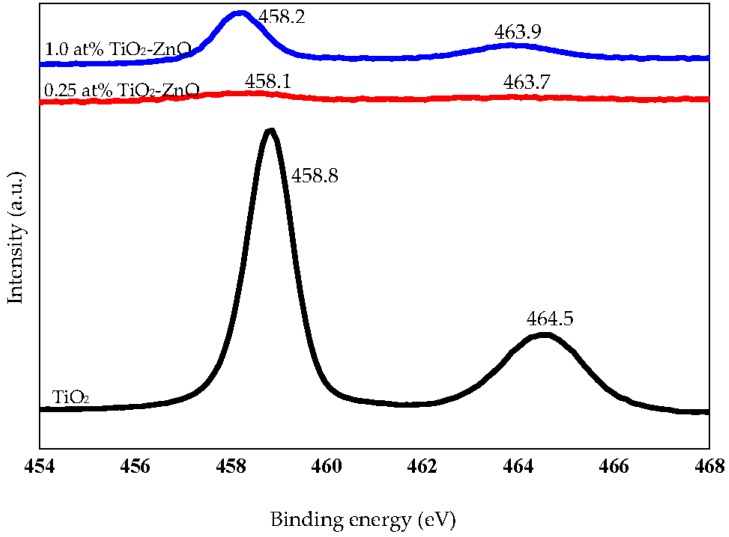
XPS spectra Ti2p of TiO_2_, 0.25 at % TiO_2_-ZnO, and 1.0 at % TiO_2_-ZnO.

The XPS spectra for Ti2p showed binding energies at 458.8 and 464.5 eV, demonstrating Ti2p_3/2_ and Ti2p_1/2_, respectively [50,51,52,53,54,55]. The Ti2p spectra indicated that the Ti in TiO_2_-ZnO were all in a Ti^4+^ state, but the heterogeneous environments of Ti^4+^ resulted in the broadening of the Ti^4+^ 2p in the XPS spectra. There is a slight decrease in the intensity of the Ti2p_3/2_ peak and a broadening of the Ti2p_1/2_ with an increase in the Ti/Zn ratio from 1.0 at % to 0.25 at % (Figure 8). This indicated a decrease in the Ti^4+^ state and the heterogeneous environment due to high intensity of ZnO as compared to TiO_2_. In addition, we can see that binding energies of TiO_2_ are shifting to lower binding energies because it has been coupled with the electron rich material ZnO (Figure 8). The existence of C1s belongs to containment carbon during calibration. A summary of XPS spectra is presented in Table 4.

### 2.3. Photoluminescence Studies

**Figure 9:**
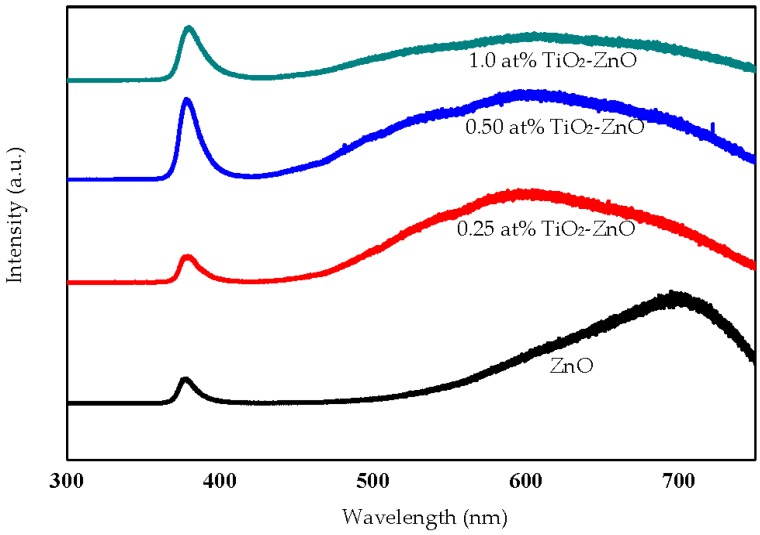
PL spectra of ZnO, 0.25 at % TiO_2_-ZnO, 0.50 at % TiO_2_-ZnO, and 1.0 at % TiO_2_-ZnO (excitation: λ = 514 nm).

Figure 9 shows the photoluminescence study for TiO_2_ loaded on ZnO nanorod film and it mainly related to some defects; for instance, zinc vacancies, zinc interstitials, oxygen vacancies, oxygen interstitials, and oxygen anti-sites. The ZnO PL spectra showed the UV emission band centered at 380 nm kept increasing with the increasing of TiO_2_-ZnO cycles. Theoretically, the refractive index of TiO_2_ (~2.55–2.9) is higher than wurtzite ZnO (~1.99). Therefore, TiO_2_ acts as antireflection layer and with the increasing of TiO_2_ cycle it may increase the absorption of light due to the oxygen anti-site at TiO_2_-ZnO interface increases the adsorption of energy and it could not be transferred to ZnO [12]. The broad visible emission band (500–800 nm) is determined by the planar defect involving twin boundaries and stacking faults of Ti and O atoms at TiO_2_-ZnO interface. The twin boundaries can be seen clearly from HRTEM image (Figure 1e). The stacking faults defect affects the PL peak at 500–800 nm range because Ti and O atoms were occupied in HCP ZnO interstices at TiO_2_-ZnO interface. Therefore, the PL peak decreased with increasing of TiO_2_ dip-coating cycle. This explanation can be supported by photocurrent density response: 0.25 at % TiO_2_-Zno was higher under solar illumination as compared to bare ZnO under UV illumination. ZnO is famous for high photo reactivity under UV illumination compared to under solar illumination. However, with the collaboration of oxygen anti-site defect, TiO_2_ adsorbed more energy and it could not be transferred to ZnO and produced lower photocurrent density and photoconversion efficiency.

### 2.4. Photoelectrochemical Response and Photoconversion Efficiency

**Figure 10:**
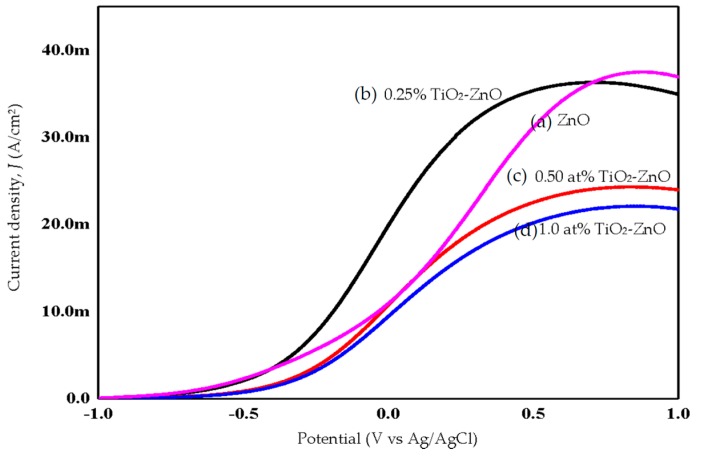
Photocurrent response of (**a**) ZnO; (**b**) 0.25% TiO_2_-ZnO; (**c**) 0.50 at % TiO_2_-ZnO; and (**d**) 1.0 at % TiO_2_-ZnO under UV illumination.

**Figure 11:**
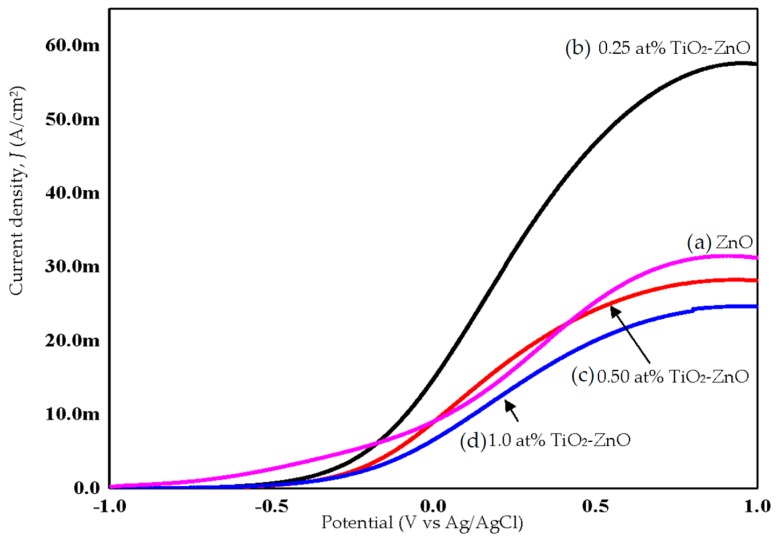
Photocurrent response of (**a**) ZnO; (**b**) 0.25 at % TiO_2_-ZnO; (**c**) 0.50 at % TiO_2_-ZnO; and (**d**) 1.0 at % TiO_2_-ZnO under solar illumination.

**Figure 12:**
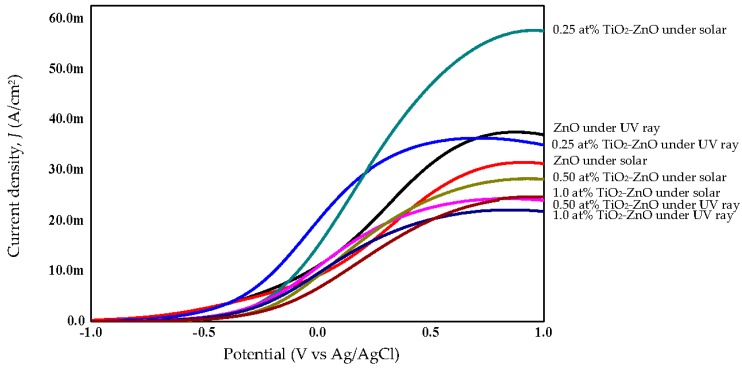
Photocurrent response for combination of all samples with respect to UV ray (300 nm) and solar illumination.

**Figure 13:**
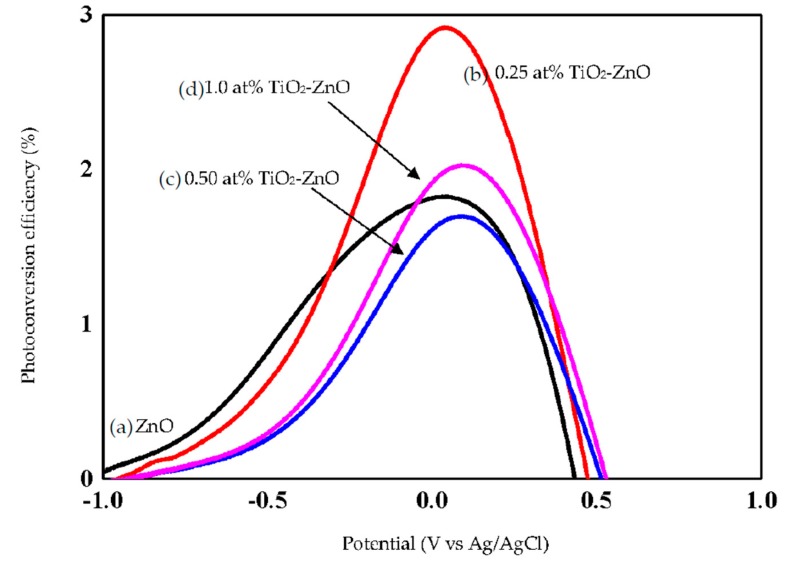
Photoconversion efficiency of (**a**) ZnO; (**b**) 0.25 at % TiO_2_-ZnO; (**c**) 0.50 at % TiO_2_-ZnO; and (**d**) 1.0 at % TiO_2_-ZnO under UV illumination.

**Figure 14:**
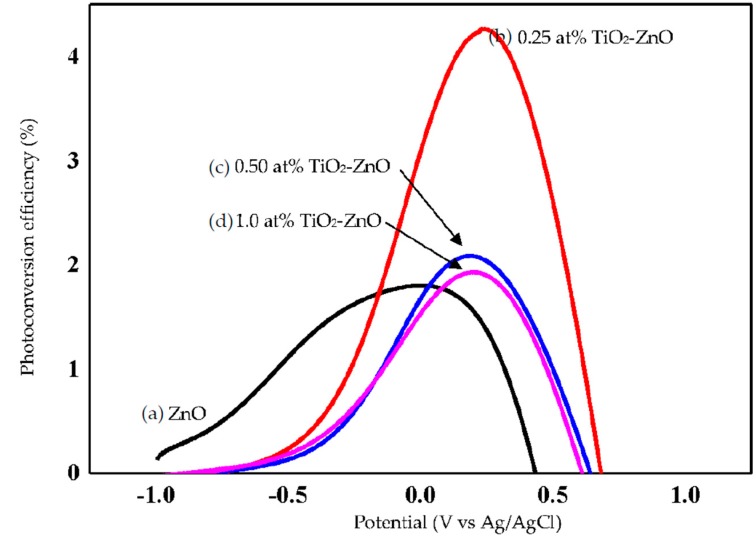
Photoconversion efficiency of (**a**) ZnO; (**b**) 0.25 at % TiO_2_-ZnO; (**c**) 0.50 at % TiO_2_-ZnO; and (**d**) 1.0 at % TiO_2_-ZnO under solar illumination.

ZnO, with its excellent electronic properties and interfacial stability, exhibited a great PEC response for hydrogen generation. The electrical simulation for water electrolysis has been studied using the PEC response, focusing on the current density analysis (Figure 10 and Figure 11). Meanwhile, Figure 12 shows combination of all photocurrent response under UV and solar illumination for better comparison. In this research work, TiO_2_-ZnO can be found on both sides of electrode. During light illumination, the active area was only 4 cm × 1 cm which was one-sided as the TiO_2_-ZnO electrode was opaque. The photoconversion efficiency (η), that is the light energy to chemical energy conversion efficiency, was subsequently calculated via Equation (1) and plotted in Figure 13 and Figure 14 [56,57], whereby 0.25 at % TiO_2_-ZnO presented the highest photocurrent density and photoconversion efficiency, regardless of whether the PEC process occurred under UV illumination or solar illumination (Figure 10b, Figure 11b, Figure 13b and Figure 14b) as compared to the sample of ZnO and samples with more than one cycle of the dip-coating process. Loading ZnO with TiO_2_ produced a photocurrent density of 19.78 mA/cm^2^ (with V vs. Ag/AgCl), as compared to ZnO 10.94 mA/cm^2^ (with V vs. Ag/AgCl) (UV illumination), 14.75 mA/cm^2^ (with V vs. Ag/AgCl) and ZnO 9.06 mA/cm^2^ (with V vs. Ag/AgCl) (solar illumination). Table 5 shows the summary of photocurrent density (mA/cm^2^) (with V vs. Ag/AgCl) for all samples under UV illumination and solar illumination. The enhancement in photocurrent density for TiO_2_ loaded on ZnO nanorod film is due to the charge-separation effect that occurred at the type-II band alignment of ZnO and TiO_2_, as discussed earlier. Meanwhile, the increase in the dip-coating cycle produced a higher amount of TiO_2_, and the electrons produced in TiO_2_ were trapped by the oxygen adsorption and could not be transferred to ZnO [37]. From PL analysis, the oxygen anti-site defect (intrinsic defect) may also give effect to the performance of TiO_2_-ZnO electrode. In Figure 12, combination of all samples showed 0.25 at % TiO_2_-ZnO exhibited highest photocurrent response regardless of whether it was under UV illumination or solar illumination. This was followed by bare ZnO, which produced good photocurrent response under both illuminations. These results support the conclusion that a small amount (0.25 at % Ti) of TiO_2_ is sufficient to produce excellent photocurrent response for PEC system. A photoconversion efficiency of ~2.9% (UV illumination) and ~4.3% (solar illumination), compared to ZnO, resulted from the presence of the Ti element in TiO_2_ loaded on ZnO nanorod film (below 1 at % Ti). Equally important, long nanorods in the presence of TiO_2_ could harvest the excited *hv* better than the ZnO and other TiO_2_ loaded samples. A high aspect ratio nanorods could absorb more *hv*, resulting in an increase in j_p_ and η [58].

(1) $$\begin{array}{cc} \eta \left(\%\right) & =\frac{\mathrm{Total}\;\mathrm{power}\;\mathrm{output}-\mathrm{electrical}\;\mathrm{power}\;\mathrm{output}}{\mathrm{Light}\;\mathrm{power}\;\mathrm{input}}\times 100\%\\ & =j_{p}\;\frac{E_{rev}^{0}-\left|E_{\mathrm{app}}\right|}{I_{0}}\times 100\% \end{array}$$

where j_p_ is the photocurrent density in mA·cm^−2^; $E_{rev}^{0}$ is the reversible potential (1.43 V Ag/AgCl); and *E*_app_ = *E*_meas_ − *E*_counter_, where *E*_meas_ is the electrical potential (V vs. Ag/AgCl) of the working electrode under illumination, and *E*_counter_ Is the electrical electrode (V vs. Ag/AgCl) of the working electrode at open circuit conditions.

### 2.5. Optical Properties

**Figure 15:**
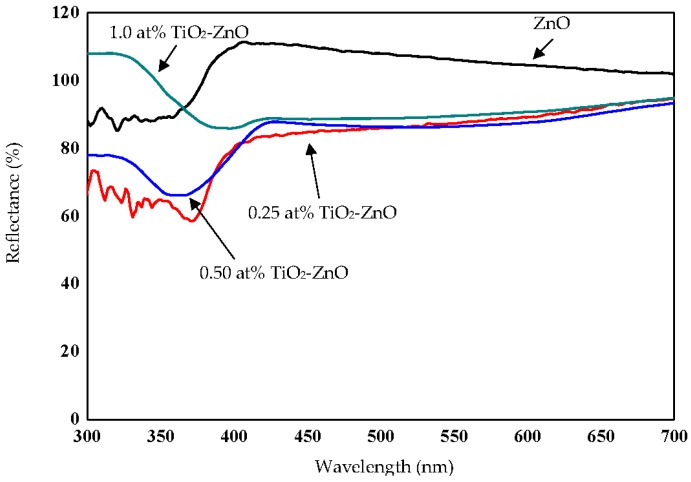
Reflectance spectra of ZnO, 0.25 at % TiO_2_-ZnO, 0.50 at % TiO_2_-ZnO, and 1.0 at % TiO_2_-ZnO.

**Figure 16:**
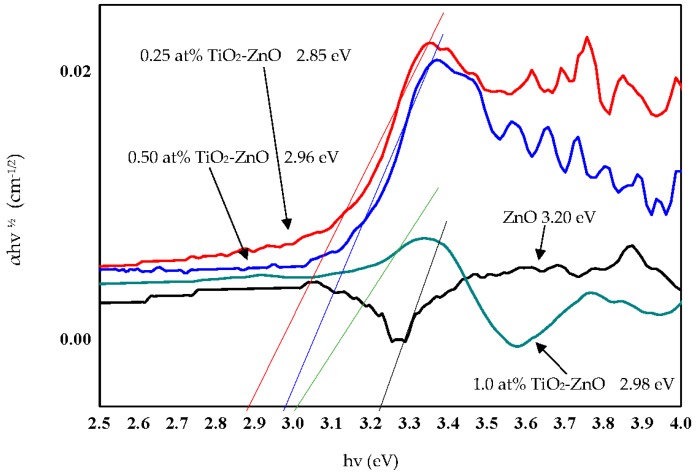
Tauc plot of ZnO, 0.25 at % TiO_2_-ZnO, 0.50 at % TiO_2_-ZnO, and 1.0 at % TiO_2_-ZnO.

**Figure 17:**
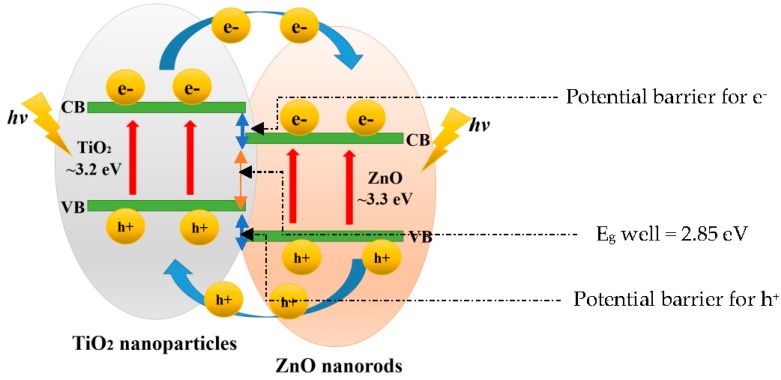
Illustration of staggered bandgap (type II) TiO_2_ loaded on ZnO nanorod semiconductor and its photo-induced charge transfer and separation.

The reflectance spectra and Tauc plot of TiO_2_ loaded on ZnO nanorod film photocatalyst based on the dip-coating cycle are plotted in Figure 15 and Figure 16. From the reflectance spectra, it showed that an increase in dip-coating cycle produced high reflection of incident light (Figure 15), and, therefore, produced low incident light absorption with increasing in dip-coating cycle. The band gap energies for ZnO, 0.25 at % TiO_2_-ZnO, 0.5 at % TiO_2_-ZnO, and 1.0 at % TiO_2_-ZnO are 3.20 eV, 2.85 eV, 2.96 eV, and 2.98 eV, respectively (Figure 16). The band gap increased with an increase in the dip-coating cycle. However, there was no large difference between the 0.5 at % and 1.0 at % TiO_2_ as shown by the photocurrent response readings. Theoretically, three types of semiconductor heterojunctions are organized by band alignment: straddling gap (type I), staggered gap (type II), and broken gap (type III). TiO_2_ loaded on ZnO nanorod film had a staggered gap (type II), as proposed by previous researchers [33,59]. TiO_2_-ZnO film exhibited band gap reduction due to the existence of planar defects [60]. Twin boundaries and stacking faults (planar defects) are correlated in band gap reduction. Based on Figure 1e, HRTEM image for TiO_2_-ZnO film, twin boundaries can clearly be seen, which will affect the heterojunction band alignment at TiO_2_ and ZnO interfaces. In addition, stacking faults defect affects band gap reduction by Ti and O atoms occupying interstices in the HCP wurtzite ZnO crystal structure. Therefore, E_g_ appears at the close contact of TiO_2_ and ZnO, and alters the electronic structure by producing resonant state (delocalized of electrons). The proposed mechanism is as follows (Figure 17), and the same mechanism has also been proposed by Hernández et al. and Fan et al. [61,62]. Electrons and holes in semiconductors are at their lowest energy states originally. Therefore, the energy gradient at the interfaces tends to spatially separate those electrons and holes that are excited by the UV illumination/solar illumination on different sides of the heterojunction. The quantum confinement effect appears at the interfaces by electrons feeling the presence of particle boundaries and responding to changes in particle size by adjusting their energy. Under illumination, the electrons are transferred from the conduction band (CB) of TiO_2_ to CB of ZnO due to the present of potential barrier for electrons (Figure 17). During the same event, the holes are transferred from the valence band (VB) of ZnO to VB of TiO_2_ with the presence of potential barriers for holes (Figure 17). The process isolates active electrons and holes and, hence, accelerates the decrease in the electron–hole pair recombination and erodes the increase in lifespan. These phenomena directly result in an intense emission quenching as revealed by the photoluminescence results (Figure 9). In addition, the high aspect ratio one-dimensional structure of the ZnO nanorods also helps to decrease the recombination probability of photogenerated carriers due to an increase in the delocalization of electrons [33,59,63].

## 3. Materials and Methods

Most researchers implemented the hydrothermal and aqueous chemical routes for ZnO formation (Table 1). However, in this research work, electrodeposition technique has been implemented for formation of ZnO. Electrodeposition technique promised better electronic performance and stronger ZnO structure [64]. This was followed by dip-coating method, which is very economical and simple.

### 3.1. The Fabrication of ZnO Nanorods

Chemicals used for the electrodeposition method for ZnO nanorods formation were 0.05 mM Zinc Chloride (ZnCl_2_), 0.1 M Potassium Chloride (KCl) and material zinc (Zn) foil (thickness 0.25 mm, 99.9% trace metals basis, Sigma-Aldrich, Saint Louis, MO, USA) under temperature 70 °C, as-prepared pH (5–6), duration 1 h, and 1 V applied potential. The electrodeposition process has been set up as a closed system of two electrodes, in which Zn foil served as cathode and platinum electrode served as anode and both electrodes were directly connected to DC power supply. The ZnO nanorods thin film has been rinsed with EMSURE ACS, 1SO, Reg. Ph Eur acetone for analysis and dried under atmosphere condition. The difference of electrodeposition method in this research work as compared to previous researches is the Zn substrate used, whereas previous researches used GaN substrate [23], Si substrate [24,65,66,67], steel substrate [25,68], FTO-coated glass [26], ITO-coated glass [6,69], and F-doped SnO_2_ coated glass [70,71,72,73,74]. In addition, electrodeposition method in this research work is template-free as compared to previous researches that used alumina membrane templates or anodic alumina template (AAM) [75,76]. Another difference of this research work electrodeposition method was seed layer-free because previous researches reported seeded substrate such as nanosheet-like Zn seed layers and ZnO seed layer [77,78]. Acid-free is an additional difference of electrodeposition method in this research work compared to previous researches. Some researchers used organic acid, for example, benzenetetracarboxylic acid, benzoic acid, and p-toluenesulfonic acid [24]. Meanwhile, citric acid is quite popular in previous electrodeposition method [27,79].

### 3.2. The Formation of TiO_2_ Nanoparticles

Chemicals used for the precipitation-peptization method were titanium (IV) isopropoxide (Sigma Aldrich, St. Louis, MO, USA, 97.0%), isopropoanol (Merck, Kirkland, QC, Canada, 99.8%), ethanol (J.Kolin, Seoul, Korea 95%) and nitric acid (merck, 65%). Solutions of nitric acid, isopropanol and deionized water were maintained in molar ratio 1:34:550 titrated with 250 mL of mixture titanium (IV) isopropoxide and isopropanol with molar ratio 1:30 under vigorous stirring for 2 h to form a white precipitate and continue stirred for another 1 h for complete hydrolyzation. The precipitate was centrifuged and washed with ethanol and white TiO_2_ gel was obtained. Subsequently, the gel was baptized in water bath with the pH 1–3 at 80 °C for 8 h until a transparent solution was obtained.

### 3.3. The Formation of TiO_2_-ZnO Composite Thin Film

The formation of TiO_2_-ZnO nanostructures composite film has been done via dip-coating method. The first step was the immersion of ZnO nanorods into TiO_2_ solution (jitter-free). Second, the ZnO nanorods remained in the TiO_2_ solution for less than five seconds and the deposition of very thin layer TiO_2_ nanoparticles occurred while it was pulled out from TiO_2_ solution. The drainage and evaporation of excess TiO_2_ solution was done by drying the dipped thin film in oven at 60 °C and calcined at 400 °C for 3 h.

### 3.4. Characterization Methods

Field Emission Scanning Electron Microscopy (FESEM) JEOL JSM-7600F (Freising, Germany) has been used to study the morphology (including the surface and cross-sectional) of TiO_2_-ZnO composite thin film. Meanwhile, elemental studies have been carried out using Hitachi Energy Dispersive X-ray Spectroscopy (EDX). The crystallinity, phase transition and photoluminescence spectra studies of TiO_2_-ZnO composite thin film were carried out via Renishaw In Via Raman microscope and supported by Bruker D8 Advance equipped with EVA-Diffract Software (Karlsruhe, Germany) X-ray Dispersive (XRD) with Cu K radiation and wavelength λ = 1.5418 Å. The photocurrent densities were obtained from photoelectrochemical cell consisted of three-electrodes (TiO_2_-ZnO nanostructures composite film (anode), platinum electrode (cathode), and Ag/AgCl in saturated KCl electrode (reference electrode)). All electrodes were immersed in 1 vol% ethylene glycol added to 1 M sodium hydroxide (NaOH). Small amount of ethylene glycol has been used as sacrificial agent during PEC procedure. Ethylene glycol worked as electron donor during PEC procedure. It supplied electron with the photogenerated VB holes for increase the electron–holes separation [80]. Light source with AM1.5 filter has been used for both UV and solar illumination (Newport model 74010) focused on the PEC cell. The light intensity was 0.652 Wcm^−2^. Meanwhile, current-applied potential was measured by using Metrohm Autolab PGSTAT204 (Herisau, Switzerland), with procedure linear sweep voltammetry potentiostatic (−1 to 1 V potential applied). The illuminated area was one-sided and, therefore, active area was 4 cm × 1 cm.

## 4. Conclusions

In summary, fine-tuning the content of TiO_2_ loaded on ZnO nanorod film is important to develop an efficient solar driven PEC system through dip-coating technique. An improvement in the photocurrent density and photoconversion efficiency was observed in the 0.25 at % TiO_2_ loaded on ZnO nanorod film with maximum value photocurrent density of 19.78 mA/cm^2^ (with V vs. Ag/AgCl) (UV illumination) and 14.75 mA/cm^2^ (with V vs. Ag/AgCl) (solar illumination) with photoconversion efficiency of ~2.9% (UV illumination) and ~4.3% (solar illumination). This finding is attributed to the excellent performance by promoting an impurity level in the binary system. In this case, the optimum 0.25 at % of TiO_2_ content acted as an electron acceptor, which was beneficial for the effective separation of the photo-induced charge carriers. However, the excessive TiO_2_ content (>0.50 at % Ti) loaded on ZnO nanorod film resulted in poor PEC performance. A suggestion for future research work is to prepare a mild TiO_2_ solution, with simple coating process in order to study the effectiveness TiO_2_-ZnO properties.

## Figures and Tables

**Table 1:** The development of hybrid TiO_2_-ZnO formation based on different approaches.

Authors	Method	Findings	Reference
Dali Shao et al. (2014)	▪ Hydrothermal (ZnO nanowires) ▪ Atomic layer deposition (TiO_2_ shell)	▪ Two steps fabrication ZnO-TiO_2_ core shell nanowires. For UV sensing application. ▪ UV illumination efficiently reduced band-to-band recombination. ▪ Maximum photoresponsivity with 495 A/W at 373 nm under −10 V.	[33]
Simelys Hernández et al. (2014)	▪ Seed layer-assisted hydrothermal route (ZnO nanowires) ▪ In situ non-acid sol–gel synthesis (TiO_2_ shell)	▪ Photocurrent densities, values of about 0.7 mA/cm^2^ under simulated solar light (AM1.5 G, 100 mW/cm^2^). ▪ The core–shell photo-anodes performance was about twice and forty- times better than the ones with a film of equivalent thickness of bare ZnO NWs and TiO NPs, respectively.	[34]
Dao et al. (2013)	▪ Hydrothermal (ZnO nanowires) ▪ Sol-gel (TiO_2_ shell)	▪ UV photodetector. ▪ Heterojunction is composed of a 5–10 nm thick p-type Cr-doped TiO_2_ nanoshell and n-type single-crystalline ZnO nanowires (50 nm radius). ▪ At a moderate reverse bias of −5 V and under UV illumination at 104 µW, it showed a switch current ratio of 140 µW and a responsivity as large as 250 A/W, while it showed nearly no response to the infrared and visible light.	[35]
Lin Lin et al. (2012)	▪ Hydrothermal method	▪ TiO_2_-ZnO n–p–n heterojunction nanorod with diameter of 30 nm. ▪ Photodegrading methyl orange has been demonstrated to increase three times compared to that of wurtzite hexagonal ZnO.	[36]
Shrabani Panigrahi et al. (2011)	▪ Aqueous chemical technique (ZnO nanorod) ▪ Solution of titanium isopropoxide [Ti (OC3 H7)_4_] followed by a heating to form the shell (TiO_2_ shell).	▪ UV sensor application. ▪ The UV photosensitivity of the nanocomposite becomes four times larger while the photocurrent decay during steady UV illumination has been decreased almost by 7 times compared to the as-grown ZnO NRs indicating high efficiency of these core–shell structures.	[37]

**Table 2:** Average compositional ratio for ZnO, one-cycle dip-coating TiO_2_-ZnO, two-cycle dip-coating TiO_2_-ZnO, and three-cycle dip-coating TiO_2_-ZnO using EDX spectroscopy analysis.

Sample	Atomic Percentage (at %)		
	Zinc	Oxygen	Titanium
ZnO	44.54	55.46	*Nil.*
One-cycle dip-coating TiO_2_-ZnO	52.95	46.80	0.25
Two-cycle dip-coating TiO_2_-ZnO	53.25	46.29	0.46
Three-cycle dip-coating TiO_2_-ZnO	61.96	36.98	1.06

**Table 3:** The average value of length, diameter and aspect ratio for ZnO, 0.25 at % TiO_2_-ZnO, 0.50 at % TiO_2_-ZnO, and 1.0 at % TiO_2_-ZnO.

Sample	Length (nm)	Diameter (nm)	Aspect Ratio
ZnO	~500	~75	6.7
0.25 at % TiO_2_-ZnO	~500	~65	7.7
0.50 at % TiO_2_-ZnO	~350	~60	5.8
1.0 at % TiO_2_-ZnO	~350	~55	6.4

**Table 4:** Summary of XPS spectra of Zn2p_3/2_, Ti2p_1/2_, Ti2p_3/2_, and O1s for samples TiO_2_, 0.25 at % TiO_2_-ZnO, 1.0 at % TiO_2_-ZnO, and ZnO.

Sample	Zn2p_3/2_	Ti2p_1/2_	Ti2p_3/2_	O1s
TiO_2_	-	464.5	458.8	530.0
0.25 at % TiO_2_-ZnO	1021.7	463.7	458.1	531.3
1.0 at % TiO_2_-ZnO	1021.5	463.9	458.2	529.7, 531.7
ZnO	1021.8	-	-	530.5

**Table 5:** The photocurrent density (mA/cm^2^) (with V vs. Ag/AgCl) of ZnO, 0.25 at % TiO_2_-ZnO, 0.50 at % TiO_2_-ZnO, and 1.0 at % TiO_2_-ZnO under UV illumination and solar illumination.

Sample	UV Illumination	Solar Illumination
ZnO	10.96	9.06
0.25 at % TiO_2_-ZnO	19.78	14.75
0.50 at % TiO_2_-ZnO	10.73	8.92
1.0 at % TiO_2_-ZnO	9.40	6.52
